# Polymorphisms in the Renin-Angiotensin System and eNOS Glu298Asp Genes Are Associated with Increased Risk for Essential Hypertension in a Mexican Population

**DOI:** 10.1155/2023/4944238

**Published:** 2023-02-17

**Authors:** Irma Isordia-Salas, David Santiago-Germán, Alejandro Flores-Arizmendi, Alfredo Leaños-Miranda

**Affiliations:** ^1^Thrombosis, Hemostasis and Atherogenesis Research Unit, H.G.R. No. 1 Dr. “Carlos Mac Gregor Sánchez Navarro”, Mexican Social Security Institute, México City, Mexico; ^2^Health Research Division, Highly Specialized Medical Unit of Traumatology, Orthopedics and Rehabilitation “Dr. Victorio de la Fuente Narváez”, Mexican Social Security Institute, Mexico City, Mexico; ^3^Hemodynamic Department, National Medical Center, Institute of Security and Social Services for State Workers, Mexico City, Mexico; ^4^Medical Research Unit in Reproductive Medicine, Highly Specialized Medical Unit No. 4, Mexican Social Security Institute, Mexico City, Mexico

## Abstract

**Background:**

Essential hypertension is the result of modifiable and genetic factors, and it is associated with increased risk for atherothrombosis. Some polymorphisms are associated with hypertensive disease. The objective was to analyze the association between eNOS Glu298Asp, MTHR C677T, AGT M235T, AGT T174M, and A1166C and ACE I/D polymorphisms with essential hypertension in the Mexican population.

**Materials and Methods:**

In the present study, 224 patients with essential hypertension and 208 subjects without hypertension were included. The Glu298Asp, C677T, M235T, T174M, A1166C, and I/D polymorphisms were determined by the PCR-RFLP technique.

**Results:**

We found statistical differences in age, gender, BMI, systolic and diastolic blood pressure, and total cholesterol between control and cases. However, we found no significant differences in HbA1c and triglycerides between both groups. We observed statistical significant differences in the genotype distribution of Glu298Asp (*P* = 0.001), I/D (*P* = 0.02), and M235T (*P* = 0.004) polymorphisms between both groups. In contrast, there were no differences related to distribution of genotypes of MTHFR C677T (*P* = 0.12), M174T (*P* = 0.46), and A1166C (*P* = 0.85) between cases and control groups.

**Conclusions:**

We identified that Glu298Asp, I/D, and M234T polymorphisms represented an increased risk for essential hypertension and those genetic variants could contribute to the presence of endothelial dysfunction and vasopressor effect, hyperplasia, and hypertrophy of smooth muscle cells, which had an impact for hypertension. In contrast, we found no association between C677C, M174T, and A1166C polymorphisms and hypertensive disease. We suggested that those genetic variants could be identified in individuals with high risk to avoid hypertension and thrombotic disease.

## 1. Introduction

Essential hypertension is determined by the combination of traditional and genetic factors and is associated with high risk for atherothrombosis [1].

Several studies demonstrated that there is a strong inherited predisposition indicating that up to 50% of the blood pressure variability is attributable to some genetic factors [2]. It has been demonstrated that some genes are associated with endothelial dysfunction, which is very important factor in the development of hypertension [3]. One of those genes is the endothelial nitric oxide synthase (eNOS), which has been demonstrated to participate in the pathophysiology of hypertension, because this enzyme is responsible for the release of nitric oxide (NO) from endothelial cells and is involved in blood pressure (BP) regulation. It has been demonstrated that the G894T (Glu298Asp) polymorphism located in the eNOS gene is associated with reduced NO synthesis and hypertension [3]. This polymorphism is associated with the modification of the structure and sequence of the protein and also modifies the functional properties of the enzyme.

In a meta-analysis published by Shi et al. [4],. an association between Glu298Asp and essential hypertension was found. Moreover, Miyamoto et al. [5] identified that this genetic variant in the eNOS gene is associated with hypertension in people from Japan. In contrast, Moe et al. [6]. demonstrated that Glu298Asp was not associated with hypertension in individuals from Singapore.

Another polymorphism associated with endothelial dysfunction is C677T localized in the gene of methylenetetrahydrofolate reductase (MTHFR), which is generated by the change of C to T, in 677 position of the nucleotide sequence, and it results in a substitution of alanine to valine amino acid. The modified enzyme is associated with higher thermolability and lower enzyme activity, which is associated with elevated blood and urinary levels of homocysteine, endothelial damage, atherosclerosis, and hypertension. Previous research by Cheng et al. [7]. demonstrated that the C677T polymorphism is associated with high blood pressure in the Chinese population.

One of the important enzymes of the renin-angiotensin system is the angiotensin-converting enzyme (ACE), which participates in the blood pressure (BP) regulation by transformation of angiotensin I to angiotensin II (AT-II) and inactivating bradykinin, which is a very strong vasodilator. Earlier studies by Rigat et al. [8] identified the I/D polymorphism in the ACE gene, characterized by insertion (I) or deletion (D) of 278 base pairs, associated with high blood pressure [9], but another investigator had failed to corroborate this result [10].

Angiotensinogen (AGT) is converted to angiotensin II by the enzyme renin, which is a potent vasoconstrictor. The M235T polymorphism is localized in the AGT gene, which results in a threonine to methionine change at position 235 and is associated with hypertension. This polymorphism is associated with higher levels of angiotensinogen in individuals with TT compared with MT or MM genotypes.

Dhanachandra et al. demonstrated that M235T polymorphism was associated with hypertension [11]. Furthermore, Procopciuc et al. [12]. corroborated that M235T represents a high risk for hypertension. However, Niu et al. failed to confirm the association [13]. Moreover, the T174M polymorphism in the AGT gene had been identified as a risk factor for hypertension [14]. Also, Mohana et al. [15]. demonstrated an association between this polymorphism and hypertension, but this association was not replicated by Kolovou et al. [16] in hypertensive individuals from Greece.

RAS participates in the regulation of electrolyte homeostasis and blood pressure control. Angiotensin II has its effect through two receptors: angiotensin II type 1 receptor (AGTR1) and angiotensin II type 2 receptor (AGT2R) [17]. It has been described a substitution of cytosine for adenine at position 1166 (A1166C) in the AT1R gene polymorphism, and this variant has been associated with hypertension [18]. Although the A1166C polymorphism has been associated with hypertension in a population from India [19] and in the Serbian population [20], this finding was not confirmed by other investigators [21, 22].

Therefore, the aim of this study was to evaluate the association of those polymorphisms, Glu298Asp, C677T, M235T, T174M, A1166C, and ACE I/D, with the increased risk of essential hypertension in the Mexican population.

## 2. Materials and Methods

### 2.1. Study Group

In a case control study, we included 224 patients with diagnosis of essential hypertension >40 and ≤70 years old (case group) and 208 individuals without hypertension (control group). Individuals were determined as hypertensive patients if they have been previously diagnosed with hypertension based on the criteria of the European Society Cardiology or if they were treated with antihypertensive drugs. Family history of coronary artery disease was defined when the subjects had a sudden death in a first-degree male relative younger than 55 years of age or a female relative younger than 65 years of age. Individuals were determined as smokers if they were currently smoking or had ceased within the last year. Dyslipidemia was defined if cholesterol level was 200 mg/dL or if the patients were treated with specific drugs. Patients and controls had not been diagnosed with type 2 diabetes mellitus or received treatment for that disease. The control group was composed of 208 individuals without history of hypertension. Data such age, sex, and history of thrombotic disease were recorded from all participants.

Demographic and clinical information was included during an interview performed by a physician, where anthropometric parameters such as body weight, height, BMI, and BP were determined. Cholesterol, triglycerides, fasting plasma glucose (FPG), and glycosylated hemoglobin (HbA1c) were determined in all participants. The polymorphisms I/D, C677T, All66C, M235T, Glu298Asp, and T174M were determined in all participants. The study protocol was approved by the Ethical and Human Committee of the Instituto Mexicano del Seguro Social and based on the Declaration of Helsinki in 1975.

### 2.2. Extraction of DNA

Genomic DNA was extracted using the QIAamp DNA Blood Mini Kit, Qiagen GmbH, Hilden Germany, following the instruction from the company.

### 2.3. Determination of eNOS Glu298Asp Genotype

As previously published, we used polymerase chain reaction (PCR) with the following primers: forward 5′CATGAGGCTCAGCCCCAGAAC-3′ and reverse 5′ AGT CAA TCC CTT TGC RGC TCAC-3′, followed by Mbo I (New England Biolabs, Beverly, MA) restriction endonuclease digestion for 8 hours at 37° C and resolution by electrophoresis on 3% agarose gel (BIO-RAD Laboratories Hercules, CA). Polymerase chain reaction was performed by denaturation at 94°C for 30 seconds (sec), annealing segment at 60°C for 30 sec, and extension at 72°C for 30 sec, repeated for 30 cycles. The resulting 206 base pair (bp) polymerase chain reaction product was cleaved into two smaller fragments of 119 and 87 bp in the presence of a T nucleotide at 894 (corresponding to Asp 298) but not in its absence (wild type). See reference [23].

### 2.4. Determination of MTHFR C667T Genotype

As previously published, we identified C677T mutation using primer forward 5′-TGAAGGAGAAGGTGTCTGC GGGA-3′ and revers 5′-AGGACGGTGCGGTGAGAGTG-3′ by PCR technique. The thermal conditions were denaturation at 94°C for 20′, annealing segment at 62°C for 20, and extension at 72°C for 20′, repeated for 30 cycles. The C677T polymorphism created a HinfI (New England Biolabs, Beverly, MA) restriction site, and the mutant allele two new fragments were 198 and 175 base pairs. See reference [23].

### 2.5. Genotyping of I/D Polymorphism

As we had previously published, the I/D polymorphism was amplified using the following primers: 5′CTGGAGACCACTCCCATCCTTTCT-3'as the forward primer and 5′ GAT GTGGCCATCACATTCGTCAGAT-3′ as the reverse primer. The presence of 190 bp fragments represented the D allele, and the presence of 490 bp fragments represented I allele. PCR was performed by denaturation at 94°C for 30 sec, annealing segment at 60°C for 30 sec, and extension at 72°C for 30 sec, repeated for 30 cycles. See reference [24].

### 2.6. Genotyping of M235T and T174M Polymorphisms

As we had previously published, we analyzed AGT-M235T and AGT-T174M using the sense primer 5′GAT GCGCACAAGGTCCTG-3′ and antisense 5′CAGGGTGCTGTCCACATGGCTCGC-3′. There was an initial denaturation at 94°C, followed by 25 cycles of one minute at 94°C, one minute at 61°C, and one minute at 72°C, followed by *Sfa*NI (New England Biolabs, Beverly Mass) restriction endonuclease digestion for 4 h at 37°C. The resulting 303 base pair (bp) polymerase chain reaction (M/M genotype) was cleaved by digestion with *SfaNI* into one smaller fragment of 303 + 266 bp in the presence of a T nucleotide (M/T) and only 266 bp fragment was present for the T/T genotype. The *AGT*-T174M genotype was determined by digestion of the same 303 bp amplified product with an existing *Nco*I cutting site The restriction fragments observed were for the 174 T/T genotype 303 bp, for the T/M genotype 303 + 211 bp + 92 bp, and for the homozygous M/M genotype 211 bp + 92 bp fragments. See reference [24].

### 2.7. Determination of ATR1 A1166C Genotype

For AT1R genotyping, the sense primer was 5′-AAAAGCCAAATCCCACTCAA-3′, and the antisense was 5′-CAGGACAAAGCAGGCTAGG-3′. The PCR thermal conditions were initial denaturation at 96°C, for 120 s, followed by 35 cycles of denaturation at 96°C for 30 s, annealing 53°C for 30 s, and extension 72°C for 60 s. PCR products were digested with 1.5 U of *DdeI* (New England Biolabs, Beverly, MA) at 37°C. The ATGR1 1166A allele results in 58 bp and 374 bp fragments, and the AT1R 1166 C allele results in 58 bp, 143 bp, and 231 bp fragments.

### 2.8. Statistical Analysis

We used percent to express categorical variables and mean ± standard deviation (SD) for continuous data. The differences between continuous variables were determined by Student's *t*-test and for categorical variables chi square test. We used the multivariate logistic regression analysis for Glu298As, I/D, M235T, A1166C, and M174T polymorphisms and hypertension as dependent variable to determinate the adjusted OR. We considered statistically significant difference with a *P* value ≤ 0.05. We used software package (SPSS 21 Inc., Chicago, IL, USA) for statistical analyses (see reference [24]).

## 3. Results

Table 1 shows the demographic data and traditional risk factors of 224 patients and 208 controls. We observed a higher percent of risk factors among patients compared to controls. We found a statistical difference (*P* < 0.001) in age between the group of patients (63.4 ± 5.2) and the control group (54.6 ± 7.3) (years). We observed a statistical difference in age, gender, BMI, glucose, cholesterol, and systolic and diastolic blood pressure between both groups. In contrast, we found no significant differences in smoking, HbA1c, and triglyceride parameters between cases and controls. Hypertensive patients and controls had no type 2 diabetes, and they had not treatment for that disease.

Table 2 shows significant differences in the genotype (OR = 2.04, 95% CI (1.36-3.15), *P* < 0.001), recessive model (OR = 2.27 (1.50-3.44), *P* < 0.001), and allele frequency (OR = 2.03, 95% CI (1.43 = 2.88), *P* < 0.001) of Glu298Asp polymorphism between both groups. Also, we observed significant differences in the frequency of the allele (*P* < 0.001) between cases and controls. As a result, individuals carrying only one Asp allele had increased risk for hypertension. The Asp allele frequency was 15.5% for cases and 18.3% for controls.

Table 3 shows the genotype distribution and allele frequency of MTHFR/C677T for both groups. Although the C677T polymorphism has been associated with hyperhomocysteinemia and endothelial dysfunction, we found no significant differences in the C677T genotype distribution (OR = 1.15, 95% CI (0.86-1.93), *P* = 0.12), recessive model (OR = 1.38 (0.87-2.17), *P* = 0.15), and allele frequency (OR = 1.25, 95% CI (0.94-1.64), *P* = 0.11) between both groups. The frequency of the T allele was 53.8% for patients and 48.3% for controls.

Table 4 represents the genotype and frequency of the ACE I/D allele between both groups. There were significant differences in the genotype (OR = 1.36, 95% CI (1.04-2.01), *P* = 0.02) or allele (OR = 1.40, 95% CI (1.06-1.86), *P* = 0.02) frequencies between both groups. The recessive model showed significant differences (OR = 1.52 (1.00-2.31), *P* = 0.04). We also identified significant difference in terms of allele frequency (*P* = 0.02). Therefore, individuals with only one D allele are with increased risk for essential hypertension. The frequencies for the D allele were 42.1% for patients and 36.8% for controls.

Table 5 shows the genotype and allele frequency of M235T polymorphism between patients and controls. We observed a positive association between this polymorphism and essential hypertension in the genotype distribution and dominant and recessive model, as well as in the allele frequency. There were significant differences in the genotype distribution (OR = 1.32, 95% CI (1.11-2.04), *P* = 0.004) and allele frequency (OR = 1.64, 95% CI (1.17-2.30), *P* = 0.003) in the M235T polymorphism between patients and controls. Also, the recessive model analysis showed statistical differences (OR = 1.54 (1.02-2.33), *P* = 0.03). Individuals carrying only one T allele could be on a higher risk for hypertension.

Table 6 represents the genotype and allele frequency of T174M between both groups. In contrast, to the other polymorphism in the angiotensinogen gene, for the T174M polymorphism, we found no significant statistical differences in the genotype distribution (OR = 1.32, 95% CI (0.84-2.01), *P* = 0.46) or allele frequency (OR = 1.24, 95% CI (0.81-1.95), *P* = 0.28). The frequency of the T allele was 13.0% (patients) and 10.6% for controls.

Table 7 represents the genotype and allele frequency of A1166C between both groups. In the present study, we found no statistical significant differences in the genotype distribution (OR = 0.98, 95% CI (0.78-1.49), *P* = 0.85) or allele frequency (OR = 1.03, 95% CI (0.73-1.46), *P* = 0.84). The recessive model showed no differences (OR = 1.43, 95% CI (0.63-3.24), *P* = 0.35). In the present study, the polymorphism A1166C (genotype or allele) was not associated with increased risk for hypertension in this group of patients.

Table 8 shows the results of multivariate logistic regression analysis. We included the variables with a *P* value ≤ 0.05 such as eNOS Glu298Asp, ACE I/D, and AGT M235T polymorphisms and traditional risk factors, and we used hypertension as the dependent variable.

## 4. Discussion

Essential hypertension is a multifactorial disease, and it has been demonstrated that it is associated with cardio- and cerebrovascular diseases [1]. Essential hypertension is produced by the combination of modifiable and genetic risk factors, but the specific mechanism is still unknown [2]. Previous studies had demonstrated that some genetic variants associated with endothelial dysfunction are also relevant to hypertensive disease, but the results are still inconclusive.

One of those polymorphisms is Glu298Asp present in endothelial nitric oxide synthase (eNOS) gene [3]. We identified that the Glu298Asp genotype (*P* < 0.001) and the 298Asp allele (*P* < 0.001) had a positive association with essential hypertension. Also, positive results were demonstrated by Nassereddine et al. [25]. who demonstrated in a case control study an association between Glu298Asp and hypertension in the adult Caucasian population from Morocco region. However, they found a lower percent of the genotype Glu/Glu 3.44% vs. 84.5% and a higher percent of genotype Asp/Asp 59.3% vs. 15.5% in the group of cases compared to our results.

Previous reports from Srivastava et al. [26] found that Glu298Asp represents a high risk for hypertension in the Asian Indian population. They included 220 cases and 200 controls. They demonstrated that the prevalence of Glu/Asp+Asp/Asp genotypes was higher in hypertensive individuals compared to the control group and association of the allele Asp with hypertension with a significant value (*P* < 0.00). However, the genotype Asp/Asp in our sample was 15.5% higher compared with the percent of that study 2.2%.

Previously, Ali and et al. [27] found a significant association between hypertension, type 2 diabetes mellitus, and high grade of obesity and 298Asp allele, compared with individuals of the control group, but without statistical significance with a significant difference. Also, Gatti et al. [28]. reported that Glu298Asp represented an independent risk factor for hypertension (*P* = 0.008) in the Brazilian population. Moreover, Shi and et al. [4] demonstrate a positive association between the Glu298Asp genetic variant and essential hypertension in Asian and Caucasian populations (*P* < 0.05). Earlier reports by Zintzaras et al. [29]. found that this polymorphism was not a risk for hypertension.

Previous studies in animal models (transgenic mice) demonstrated that overexpression of eNOS is associated with lower blood pressure. In contrast, the inhibition of eNOS gene in normal individuals was associated with lower levels of nitric oxide, high levels of blood pressure, and endothelial dysfunction [30].

Earlier studies demonstrated that eNOS containing a sequence Asp 298 in the protein is associated with selective proteolytic cleavage in endothelial and vascular cells [31], which is associated with lower nitric oxide enzyme activity, and it has been demonstrated that it also affects eNOS localization to the caveolar membrane [32]. Nitric oxide has a vasorelaxant action, which is involved in the regulation of blood pressure. Moreover, nitric oxide has several functions such as inhibition of aggregation of platelets, leukocyte, and platelet adhesion inhibition and muscle cell migration and proliferation. It has been demonstrated that lower nitric oxide production is associated with endothelial dysfunction and thrombotic disease.

eNOS is constitutively expressed but can also be inducible through diverse regulations at transcriptional and posttranscriptional levels, and such modifications may have an impact on vascular regulation and atherothrombotic process.

Methylenetetrahydrofolate reductase (MTHFR) is an enzyme that plays a very important role in the homocysteine and folate metabolism. The mutation C677T is present in the MTHFR gene and is associated with high thermolability and decreases enzymatic activity [7]. Subjects with this mutation have higher levels of homocysteine due to the absence of conversion of homocysteine to methionine by this enzyme. Higher levels of homocysteine are associated with cardiovascular disease and hypertension [7]. We found no association between the C677T polymorphism and essential hypertension (*P* = 0.90) in this group of patients. Also, negative results were found by Fowdar et al. [33]. who documented that C667T was not associated with essential hypertension in Caucasian Australian individuals. Also, Amrani et al. [34]. demonstrated in an Algerian population a negative association between the C677T polymorphism and essential hypertension (*P* = 0.07). In contrast, Cheng et al. [7]. proposed that the 677TT genotype is associated with hypertension in individuals from China. Moreover, Heux et al. [35]. found in a case control study that the C677T mutation represented an increased risk for hypertension after adjusting for body mass index (*P* = 0.03).

Hypertension is a multifactorial disease, and its etiology is still not well known. Some factors had been described to be involved in the pathogenesis of this disease such as sodium intake, body mass index, smoking, and genetics factors.

Several studies had demonstrated an important role of genetic background is in the development and treatment of hypertension, which has been demonstrated with some therapeutic drug resistance.

We had previously documented that the I/D genetic variant represents a high risk for thrombotic disease such idiopathic ischemic stroke [24], but not for cardiovascular disease [36] in young Mexican patients. In the present study, the polymorphism I/D was associated with higher risk for hypertension. There was a statistical differences in genotype distribution (*P* = 0.02) and allele frequency (*P* = 0.02) between case and control groups. Also, positive results were obtained by Hadian et al. [37] in Pakistani hypertensive individuals. Previously, Li et al. [9] demonstrated that I/D was associated with hypertensive disease. However, Vassilikioti et al. [10] found no association between I/D variant and hypertension. Also, Martinez et al. [38]. demonstrated that I/D polymorphism was not associated with this disease. However, they identified increased ACE activity in Spanish patients with I/D or D/D genotype.

In a meta-analysis by Liu et al. [39], it was demonstrated in 57 studies with more than 38,000 hypertensive individuals that D allele was associated with increased risk for essential hypertension. They determined an increased susceptibility in allelic, homozygote, dominant, or regressive model analysis associated with increased risk for the disease.

Endothelial dysfunction had been associated with the presence of Glu298Asp, C677T, or I/D polymorphism which could be related to a malfunction of the molecular cross-talk between the nucleus and the mitochondria [28].

Clinical studies had identified that M235T polymorphism in the angiotensinogen gene was associated with higher risk for hypertension [11]. We observed a significant statistical difference in the genotype (*P* < 0.005) and allele frequency (*P* = 0.003) of the M235T polymorphism between hypertensive patients and controls. Also, positive results were found by Dhanachandra et al. [11] in hypertensive patients. Also, Procopciuc et al. [12]. showed that M235T represented an increased risk for hypertension in the Romanian population, and it was confirmed by Agachan et al. [40]. in Turkish patients. Moreover, Hata et al. [41]. demonstrated a positive correlation between the M235T polymorphism and hypertension in a population from Japan. Also, Kim and et al. [42] demonstrated that the heterozygous (M/M) and the M allele of M235T polymorphism represented risk factors for hypertension in the Korean population.

In contrast, Martinez et al. [33]. identified similar genotype M235T distribution between cases and controls in the Spanish population. Also, no association was found by Niu et al. [13] in Chinese hypertensive individuals. We had previously explored the association between the M235T polymorphism and atherothrombotic disease in our population. We demonstrated that M235T was associated with myocardial infarction [36] and stroke [24] in young Mexican individuals.

Another polymorphism in the AGT gene is M174T, and it had been associated with increased blood pressure [14]. Our results demonstrated no differences in the distribution of the genotype distribution (*P* = 0.28) and the frequencies of the allele T frequency (*P* = 0.30) between both groups. Also, negative results were reported by Kolovou et al. [16]. in Greek hypertensive individuals. In contrast, Liao and et al. [43] identified that the polymorphism T174M was associated with hypertension. Furthermore, in another meta-analysis, Mohana et al. [15] reported an association between T174M and essential hypertension. We had previously reported that polymorphism AGT T174M was associated with idiopathic ischemic stroke [24], but this polymorphism was not associated with cardiovascular disease [36] in young Mexican population.

Our results suggest that individuals carrying one or more polymorphism associated with endothelial dysfunction and/or renin-angiotensin system alterations combined with other environmental risk factors could be in higher risk for hypertension, stroke, or cardiovascular disease even at a young age, as this was also reported by Szolnoki et al. [44]. in the Caucasian population. They found that combination of genotypes Glu/Asp or Asp/Asp with homozygous 677TT or homozygous DD was associated with higher risk for cerebrovascular disease.

There was no difference related to triglycerides or HbA1c between both groups. In contrast, we identified differences in terms of glucose and total cholesterol between both groups. Therefore, we consider that it is very important that hypertensive patients had a good control of metabolic parameters of diabetes and dyslipidemia in order to avoid the atherothrombotic disease development.

One limitation of the study was the number of participants (patients and controls). Another limitation was that the groups were not matched by sex and age. The study has some strengths such as all participants had the same ethnic background and all samples were analyzed by the same individual.

## 5. Conclusions

In the present study, we identified that eNOS Glu298Asp, AGT M234T, and ACE I/D polymorphisms were associated with essential hypertension generated probably by the presence of endothelial dysfunction and vasopressor effect, hyperplasia, and hypertrophy of smooth muscle cells. In contrast, MTHFR C677T, AGT T174M, and AGTR1 A1166C did not represent a higher risk for hypertensive disease. Our results consider that combination of genetic and some traditional risk factors in each individual could determine the development of essential hypertension, which could contribute for the risk of atherothrombotic development. We suggest that those genetic variants be identified in individuals with high risk to avoid or diminish hypertension thrombotic disease. Also, hypertensive patients should have a good control of blood pressure and metabolic parameters. More studies with higher number of patients and controls matched by sex and gender could be desirable to confirm our findings.

## Figures and Tables

**Table 1:** Demographic and clinical characteristics between hypertensive patients and controls.

	Patients (224)	Controls (208)	*P* value^∗^
Age (years) (mean ± SD)	63.4 ± 5.2	54.6 ± 7.3	<0.0001
Female, *n* (%)	154 (68.7)	136 (58.7)	0.001
*Clinical*			
BMI (kg/m^2^)	29.2 ± 6.4	28.7 ± 3.7	0.04
SPB (mmHg)	138 ± 8	113 ± 11	<0.001
DPB (mmHg)	74 ± 6	76 ± 8	<0.001
Dyslipidemia, *n* (%)	98 (43.7)	84 (40.3)	0.01
Smoking, *n* (%)	48 (21.4)	51 (24.5)	NS
*Biochemical*			
FPG (mg/dL)	91 ± 5.8	84 ± 7.3	0.01
HbA1c (%)	5.7 ± 0.4	5.4 ± 0.8	NS
Total cholesterol (mg/dL)	194 ± 21	182 ± 28	0.01
Triglycerides (mg/dL)	142.4 ± 52.6	140 ± 37.4	NS

BMI: body mass index; SBP: systolic blood pressure; DBP: diastolic blood pressure; FPG: fasting plasma glucose; HbA1c: glycated hemoglobin; SD: standard deviations; NS: no significance.

**Table 2:** eNOS Glu298Asp genotype and allele frequency in patients with essential hypertension and controls.

	Patients (*n* = 224)	Controls (*n* = 208)	*P* value^∗^	OR (95% CI)
*Genotype*				
Glu/Glu, *n* (%)	114 (50.8)	146 (70.2)	0.001^∗^	OR 2.04 (1.36–3.15)
Glu/Asp, *n* (%)	96 (42.9)	58 (27.9)		
Asp/Asp, *n* (%)	14 (6.3)	4 (1.9)		
*Dominant model*				
Asp/Asp *vs.* Glu/Asp+Glu/Glu, *n* (%)	14 *vs.*96 + 114	4 + 58*vs.* 146	0.02^∗^	OR = 3.40 (1.02–12.45)
*Recessive model*				
Glu/Glu *vs.* Glu/Asp+Asp/Asp, *n* (%)	114 *vs.*96 + 14	146 *vs.*58 + 4	0.001^∗^	OR = 2.27 (1.50–3.44)
*Allele frequency*				
Glu, *n* (%)	324 (84.5)	350 (81.7)	0.001^∗^	OR = 2.03 (1.43–2.88)
Asp, *n* (%)	124 (15.5)	66 (18.3)		

Data presented are number and % of patients. CI: confidence interval; odds ratio, ^∗^*X*^2^.

**Table 3:** MTHFR C677T genotype and allele frequency in patients with essential hypertension and controls.

	Patients (*n* = 224)	Controls (*n* = 208)	*P* value^∗^	OR (95% CI)
*Genotype*				
C/C, *n* (%)	51 (22.8)	60 (28.8)	0.12^∗^	OR = 1.15 (0.86–1.93)
C/T, *n* (%)	105 (46.8)	95 (45.7)		
T/T, *n* (%)	68 (30.4)	53 (25.5)		
*Dominant model*				
T/T *vs.* C/T+C/T, *n* (%)	68 *vs.*105 + 51	53 *vs*. 95 + 60	0.26^∗^	OR = 1.27 (0.82–1.99)
*Recessive model*				
C/C *vs.* C/T+T/T, *n* (%)	51 *vs.*105 + 68	60 *vs*. 95 + 53	0.15^∗^	OR = 1.38 (0.87–2.17)
*Allele frequency*				
C, *n* (%)	207 (46.2)	215 (51.7)	0.11^∗^	OR = 1.25 (0.94–1.64)
T, *n* (%)	241 (53.8)	201 (48.3)		

Data presented are number and % of patients. CI: confidence interval; odds ratio, ^∗^*X*^2^.

**Table 4:** ACE I/D genotype and allele frequency in patients with essential hypertension and controls.

	Patients (*n* = 224)	Controls (*n* = 208)	*P* value^∗^	OR (95% CI)
*Genotype*				
I/I, *n* (%)	68 (30.4)	83 (39.9)	0.02^∗^	1.36 (1.04–2.01)
I/D, *n* (%)	112 (50.0)	98 (47.1)		
D/D, *n* (%)	44 (19.6)	27 (13.0)		
*Dominant model*				
D/D *vs.* I/D+D/D, *n* (%)	44 *vs.*112 + 68	27 *vs.*98.+83	0.02^∗^	1.92 (1.08–3.44)
*Recessive model*				
I/I *vs.* I/D+D/D, *n* (%)	68 *vs.*112 + 44	83 *vs*. 98 + 23	0.04^∗^	1.52 (1.00–2.31)
*Allele frequency*				
I, *n* (%)	248 (57.9)	264 (63.2)	0.02^∗^	1.40 (1.06–1.86)
D, *n* (%)	200 (42.1)	152 (36.8)		

Data presented are number and % of patients. CI: confidence interval; odds ratio, ^∗^*X*^2^.

**Table 5:** AGT M235T genotype and allele frequency in patients with essential hypertension and controls.

	Patients (*n* = 224)	Controls (*n* = 208)	*P* value^∗^	OR (95% CI)
*Genotype*				
M/M, *n* (%)	128 (37.1)	140 (67.3)	0.004^∗^	1.32 (1.11–2.04)
M/T, *n* (%)	72 (32.1)	60 (28.9)		
T/T, *n* (%)	24 (10.8)	8 (3.8)		
*Dominant model*				
T/T *vs.* M/T+T/T, *n* (%)	24 *vs.*72 + 128	8 *vs*. 60 + 140	0.006^∗^	3.0 (1.25–7.45)
*Recessive model*				
M/M *vs.* M/T+T/T, *n* (%)	128 *vs.*72 + 24	140 *vs*. 60 + 8	0.03^∗^	1.54 (1.02–2.33)
*Allele frequency*				
M, *n* (%)	328 (73.2)	340 (81.75)	0.003^∗^	1.64 (1.17–2.30)
T, *n* (%)	120 (26.8)	76 (18.25)		

Data presented are number and % of patients. CI: confidence interval; odds ratio, ^∗^*X*^2^.

**Table 6:** AGT T174M genotype and allele frequency in patients with essential hypertension and controls.

	Patients (*n* = 224)	Controls (*n* = 208)	*P* value^∗^	OR (95% CI)
*Genotype*				
T/T, *n* (%)	170 (75.9)	166 (79.8)	0.46^∗^	1.32 (0.84–2.01)
T/M, *n* (%)	50 (22.3)	40 (19.2)		
M/M, *n* (%)	4 (1.8)	2 (1.0)		
*Dominant model*				
T/T *vs.* T/M+M/M, *n* (%)	170 *vs.*50 + 4	166 *vs*. 40 + 2	0.33^∗^	1.26 (0.78–2.03)
*Recessive model*				
M/M *vs.* T/M+M/M, *n* (%)	4 *vs*. 50 + 170	2 *vs*. 40 + 166	0.44^∗^	1.87 (0.29–14.86)
*Allele frequency*				
M, *n* (%)	390 (87.0)	372 (89.4)	0.28^∗^	1.24 (0.81–1.95)
T, *n* (%)	58 (13.0)	44 (10.6)		

Data presented are number and % of patients. CI: confidence interval; odds ratio, ^∗^*X*^2^.

**Table 7:** AGTR1 A1166C genotype and allele frequency in patients with essential hypertension and controls.

	Patients (*n* = 224)	Controls (*n* = 208)	*P* value^∗^	OR (95% CI)
*Genotype*				
A/A, *n* (%)	148 (66.1)	135 (64.9)	0.85^∗^	0.98 (0.78–1.49)
A/C, *n* (%)	58 (25.9)	61 (29.3)		
C/C, *n* (%)	18 (8.0)	12 (5.8)		
*Dominant model*				
A/A *vs*. A/C+C/C, *n* (%)	148 *vs.*58 + 18	135 *vs*. 61 + 12	0.87^∗^	0.97 (0.64–1.47)
*Recessive model*				
C/C *vs.* A/C+A/A, *n* (%)	18 *vs.*58 + 148	12 *vs*. 61 + 135	0.35^∗^	1.43 (0.63–3.24)
*Allele frequency*				
A, *n* (%)	354 (79.0)	331 (79.6)	0.84^∗^	1.03 (0.73–1.46)
C, *n* (%)	94 (21.0)	85 (20.4)		

Data presented are number and % of patients. CI: confidence interval; odds ratio, ^∗^*X*^2^.

**Table 8:** Multiple logistic regression analysis using essential hypertension as the dependent variable.

Risk factor	Adjusted OR (95% CI)	*P* value^∗^
Age	1.02 (1.0–2.6)	0.02
BMI	1.13 (1.0–2.18)	0.05
FG	1.27 (1.1–2.12)	0.04
Glu298Asp	2.37 (1.4–3.12)	0.001
I/D	1.44 (1.9–2.82)	0.02
M235T	1.24 (1.1–2.43)	0.004

*χ* ^2^ test multivariate logistic regression. OR: odds ratio; BMI: body mass index; FG: fasting glucose.

## Data Availability

Access to data is restricted because of patients' privacy.
